# Case Report: Novel *NIPBL* Variants Cause Cornelia de Lange Syndrome in Chinese Patients

**DOI:** 10.3389/fgene.2021.699894

**Published:** 2021-07-30

**Authors:** Ying Peng, Changbiao Liang, Hui Xi, Shuting Yang, Jiancheng Hu, Jialun Pang, Jing Liu, Yingchun Luo, Chengyuan Tang, Wanqin Xie, Hua Wang

**Affiliations:** ^1^Department of Medical Genetics, National Health Commission Key Laboratory of Birth Defects for Research and Prevention, Hunan Provincial Maternal and Child Health Care Hospital, Changsha, China; ^2^Department of Health Care, Hunan Provincial Maternal and Child Health Care Hospital, Changsha, China; ^3^Department of Nephrology, Hunan Provincial Key Laboratory of Kidney Disease and Blood Purification, The Second Xiangya Hospital of Central South University, Changsha, China

**Keywords:** Cornelia de Lange syndrome, *NIPBL*, whole-exome sequencing, SNP array, prenatal diagnosis

## Abstract

Cornelia de Lange syndrome (CdLS) is a genetic disorder characterized by multisystemic malformations. Mutation in the *NIPBL* gene accounts for nearly 60% of the cases. This study reports the clinical and genetic findings of three cases of CdLS from unrelated Chinese families. Clinically, all the three cases were classified as classic CdLS based on the cardinal (distinctive facial features and limb malformations) and suggestive (developmental delay, growth retardation, microcephaly, hirsutism, etc.) manifestations. SNP array detected a novel *de novo* heterozygous microdeletion of 0.2 Mb [arr[GRCh37]5p13.2(36848530_37052821) × 1] that spans the first 43 exons of *NIPBL* in the fetus with nuchal translucency thickening in case 1. Whole-exome sequencing in family trios plus Sanger sequencing validation identified a *de novo* heterozygous *NIPBL* c.5566G>A (p.R1856G) mutation in the fetus with intrauterine growth retardation in case 2 and a novel *de novo* heterozygous *NIPBL* c.448dupA (p.S150Kfs^*^23) mutation in the proband (an 8-month-old girl) in case 3. The cases presented in this study may serve as references for increasing our understanding of the mutation spectrum of *NIPBL* in association with CdLS.

## Introduction

Cornelia de Lange syndrome (CdLS, OMIM #122470, 300590, 610759, 300882 and 614701) is a genetically heterogeneous congenital multisystemic disorder. The typical clinical manifestations include distinctive craniofacial features (finely arched eyebrows, synophrys, long eyelashes, low-set posteriorly rotated ears, long philtrum, thin upper lip, and depressed nasal bridge), growth retardation, behavioral abnormality, and upper limb deficiencies (Boyle et al., 2015). The features of this disorder vary widely among affected individuals and range from relatively mild to severe. The prevalence of CdLS is estimated to be between 1:10,000 and 1:50,000 live births (Barisic et al., 2008; Mannini et al., 2013).

Approximately 60% of patients with CdLS have a disease-causing mutation in the *NIPBL* (OMIM #608667) gene, the human homolog of the *Drosophila* Nipped-B gene, which plays important roles in mitotic sister chromatid cohesion and in regulation of other genes. About 10% of cases are caused by mutations in the other four genes belonging to the cohesion pathway, including *SMC1A, SMC3, HDAC8*, and *RAD21*. The underlying genetic cause of the remaining 30% of cases is unknown (Mannini et al., 2013; Boyle et al., 2015).

CdLS is usually diagnosed by postnatal clinical findings (Kline et al., 2018). Prenatal diagnosis of CdLS could be made in terms of the presence of congenital diaphragmatic hernia, characteristic limb abnormalities, and facial profile as detected by fetal ultrasound. However, routine prenatal ultrasonography was reported to fail to detect over two-thirds of cases of CdLS with major malformations (Barisic et al., 2008; Avagliano et al., 2017).

In this study, we report the clinical and genetic findings of three cases of Chinese patients with CdLS in the setting of prenatal diagnosis.

## Case Presentation

### Case 1

**Figure 1:**
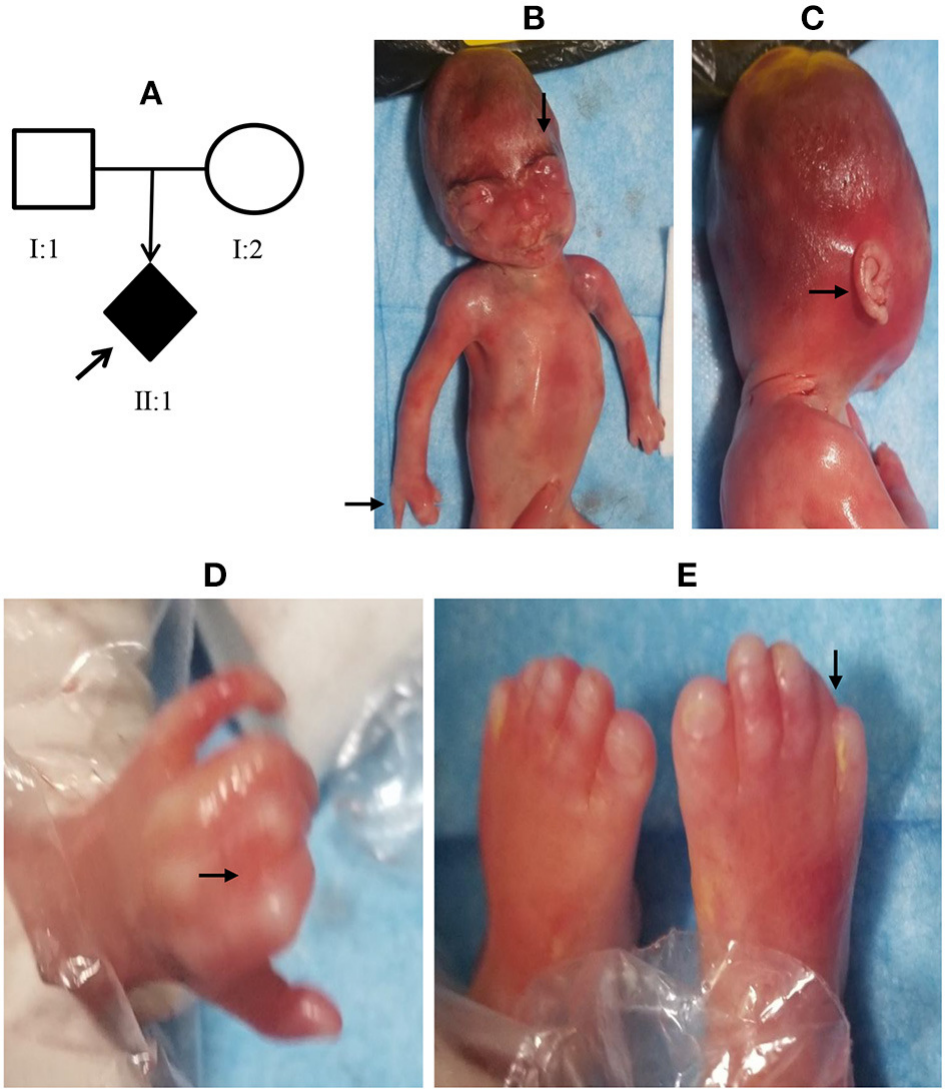
Facial features and limb malformations of the fetus in case 1. A 28-year-old Chinese woman was referred for prenatal diagnosis at 16 weeks of gestation due to fetal nuchal translucency (NT) thickening **(A)**. The pregnancy was terminated at 20 weeks, and autopsy showed distinct facial features **(B, C)** and limb defects **(B, D, and E)** in the fetus as indicated by the arrows.

A 28-year-old Chinese woman, gravidity 1, parity 0, was referred to our institution for prenatal diagnosis at 16 weeks of gestation due to fetal nuchal translucency (NT) thickening (NT, 4.8 mm; crown–rump length: 58 mm) as detected by ultrasound examination at 12 weeks and 3 days of gestation (Figure 1A). The first-trimester screening showed a low risk for Down syndrome. Known family histories of genetic diseases and exposure to teratogenic agents were denied. G-banded (320–400 bands) karyotyping showed a normal chromosomal karyotype (46,XY) in the fetus. SNP array in family trios revealed a *de novo* heterozygous microdeletion of 0.2 Mb at chromosome 5p13.2 [arr[1GRCh37]5p13.2 (36848530_37052821) × 1] in the fetus (Supplementary Figure 1). This gross deletion spans the first 43 exons of the 47 exons of the *NIPBL* gene as visualized by USUC genome browser (http://genome-asia.ucsc.edu/) and had not been documented previously. Real-time quantitative PCR using primer pairs specifically targeting exons 26, 29, and 30 of *NIPBL*, respectively, confirmed the *de novo* deletion in the fetus (Supplementary Figure 2). The haplodeficiency of *NIPBL* had not been evaluated by the Clingen Dosage Sensitivity group. *NIPBL* has a high pLI (probability of being loss-of-function intolerant) of 1 in gnomAD (http://gnomad.broadinstitute.org/) and a high rank HI (probability of being a haploinsufficient gene) of 3.01% in Decipher (https://www.deciphergenomics.org/). Thus, a point value of 0.15 was assigned according to the ACMG guidelines of CNV interpretation (Riggs et al., 2020). *De novo* loss-of-function (nonsense or frameshift) mutations were confirmed in more than six families (Gillis et al., 2004), and thereby, a point value of 0.9 was assigned. In addition, segregation of CdLS with a donor splice site of *NIPBL* in a single family with four affected siblings was reported (Slavin et al., 2012), and thus, a score of 0.15 points was assigned. Taken together, a final score of 1.2 points reached the threshold of pathogenicity (>0.99), and this CNV was classified as pathogenic and considered as a causative mutation for CdLS. The pregnancy was terminated at 20 weeks of gestation. The distinctive facial features and limb malformations of the male fetus were documented at autopsy (case 1 in Table 1; Figure 1), which support a diagnosis of classic CdLS (Supplementary Table 1) in terms of the clinical classification guidelines (Kline et al., 2018).

**Table 1:** Comparison of the three cases of Cornelia de Lange syndrome (CdLS).

**Parameter**	**Case 1**	**Case 2**	**Case 3**
Age and pregnancy history	28 years, para 1-0-0-0	36 years, para 2-1-0-1	20 years, para 2-1-0-1
Prenatal diagnostic indications	Nuchal translucency (NT) thickening at 12^+3^ weeks	Fetal growth restriction (FGR) found at 31^+2^ weeks	First child (an 8-month-old girl) affected
Gestation weeks at referral	16 weeks	34 weeks	17 weeks
Genetic tests	Karyotyping, SNP array, real-time PCR	Karyotyping, SNP array, WES in trios. Sanger sequencing	WES in trios, Sanger sequencing
Genetic variation	*De novo* heterozygous arr[1GRCh37]5p13.2 (36848530_37052821) × 1 in the fetus	*De novo* heterozygous *NIPBL* c.5566A>G in the fetus	*De novo* heterozygous *NIPBL* c.448dupA in the proband
Amino acid alteration	Truncation of 1–43 exons of NIPBL	p.R1856G	p.S150Kfs*23
Pregnancy outcome	Termination at 20 weeks	Natural delivery at 37^+3^ weeks	Continue pregnancy
Clinical features	Low hairline, “penciled in” eyebrows without synophrys, long philtrum, thin lips, and low-set posteriorly rotated ears; limb defects included micromelia, split hand malformation of the right hand with oligodactyly, clinodactyly in the left hand, and syndactyly of toes 4 and 5 of the right foot	Low hairline, hypertrichosis of the eyebrows, broad depressed nasal bridge, smooth long philtrum with thin lips, short and broad neck, hearing loss screening fail, weak crying, and feeding difficulty	Excessive body hair, synophry, hypertrichosis of the eyebrows, long eyelashes, broad depressed nasal bridge, turned angles of the mouth with thin lips, short neck and high-arched palate, short fingers and toes, split hand malformation of the right hand and proximally placed thumb and the fifth finger (curved inward) of the left hand
Follow-up	/	Physical examination 2 weeks after birth	Ultrasound examination at 24 weeks

### Case 2

**Figure 2:**
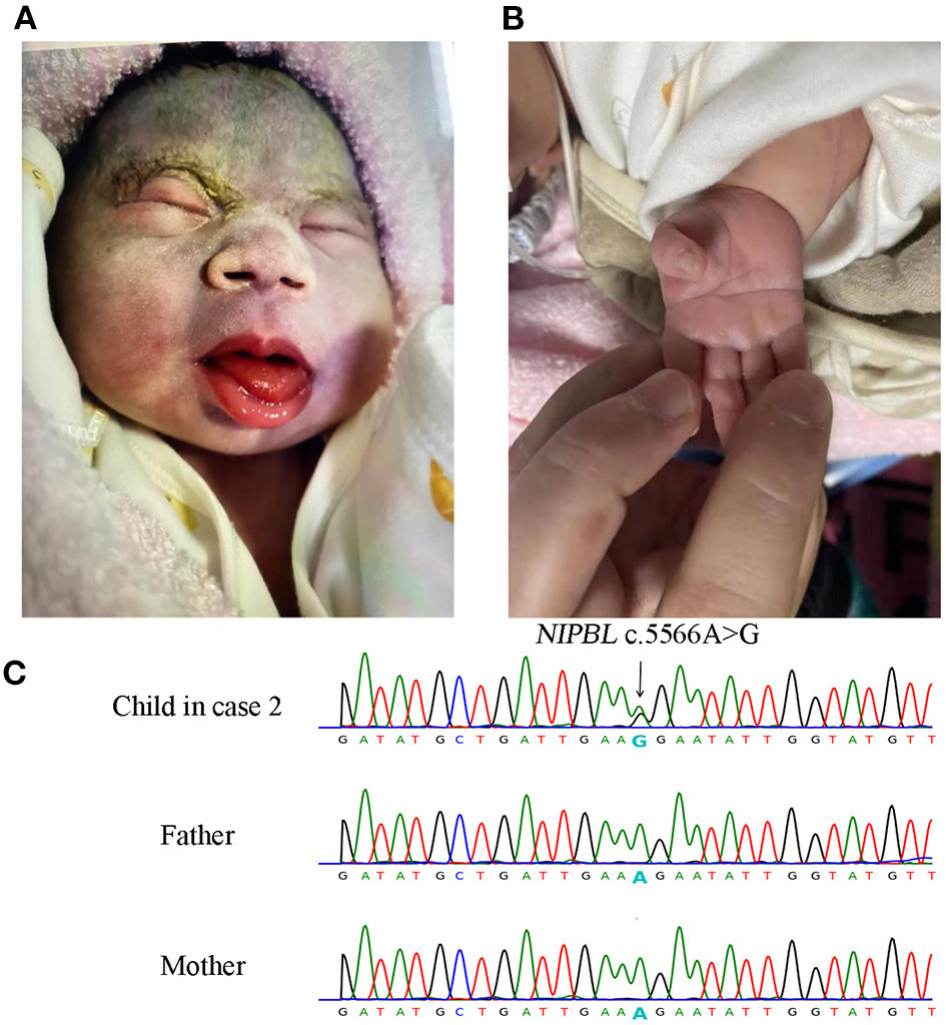
Facial and limb features of the newborn and Sanger sequencing results of *NIPBL* c.5566A>G mutation in case 2. The newborn presented with low hairline, hypertrichosis of the eyebrows, broad depressed nasal bridge, smooth long philtrum with thin lips **(A)**, and single palmar crease **(B)**. Sanger sequencing validated the *de novo* c.5566A>G mutation in the newborn **(C)**.

A 36-year-old Chinese woman, gravidity 2, parity 1, negative family and past histories of genetic diseases, was referred to our institution at 34 weeks of gestation because of ultrasound finding of fetal growth restriction (FGR) at 31 weeks and two days of gestation. Ultrasound examination revealed a biparietal diameter of 72 mm [−1.43 standard deviation (SD)], head circumference 263 mm (−2.47 SD), abdominal circumference 249 mm (10th percentile), femur length 55 mm (−2.1 SD), and estimated fetal body weight of 1,327 g (first percentile). No other major anomalies were found. The maternal serum screening at the second trimester suggested a high risk for Down syndrome, but the non-invasive prenatal testing (NIPT) indicated a low risk. The baby was naturally delivered at 37 weeks and three days of gestation before the genetic tests were completed, with a body weight of 2,300 g, length of 42.0 cm, and head circumference of 29 cm (<−3 SD) (Supplementary Figure 3). There was no birth asphyxia, and the Apgar score was 10/10. The clinical features of the newborn suggested a diagnosis of classic CdLS (Figures 2A,B; Table 1; Supplementary Table 1). The baby had a normal karyotype (46,XX) and carried no common pathogenic variants as revealed by SNP array (data not shown). A non-synonymous variant in *NIPBL* (NM_133433.4: exon29: c.5566A>G: p.R1856G) was identified by using WES, and this *de novo* variation was further confirmed by using Sanger sequencing (Figure 2C). In the search of databases, this mutation has been indexed in Clinvar (accession No. VCV000099934.1) and associated with CdLS. However, the detailed clinical phenotypes had not been noted regarding this mutation. Our case for the first time provided details on clinical features of CdLS caused by the c.5566A>G variation, adding more evidence to the pathogenicity of this mutation.

### Case 3

**Figure 3:**
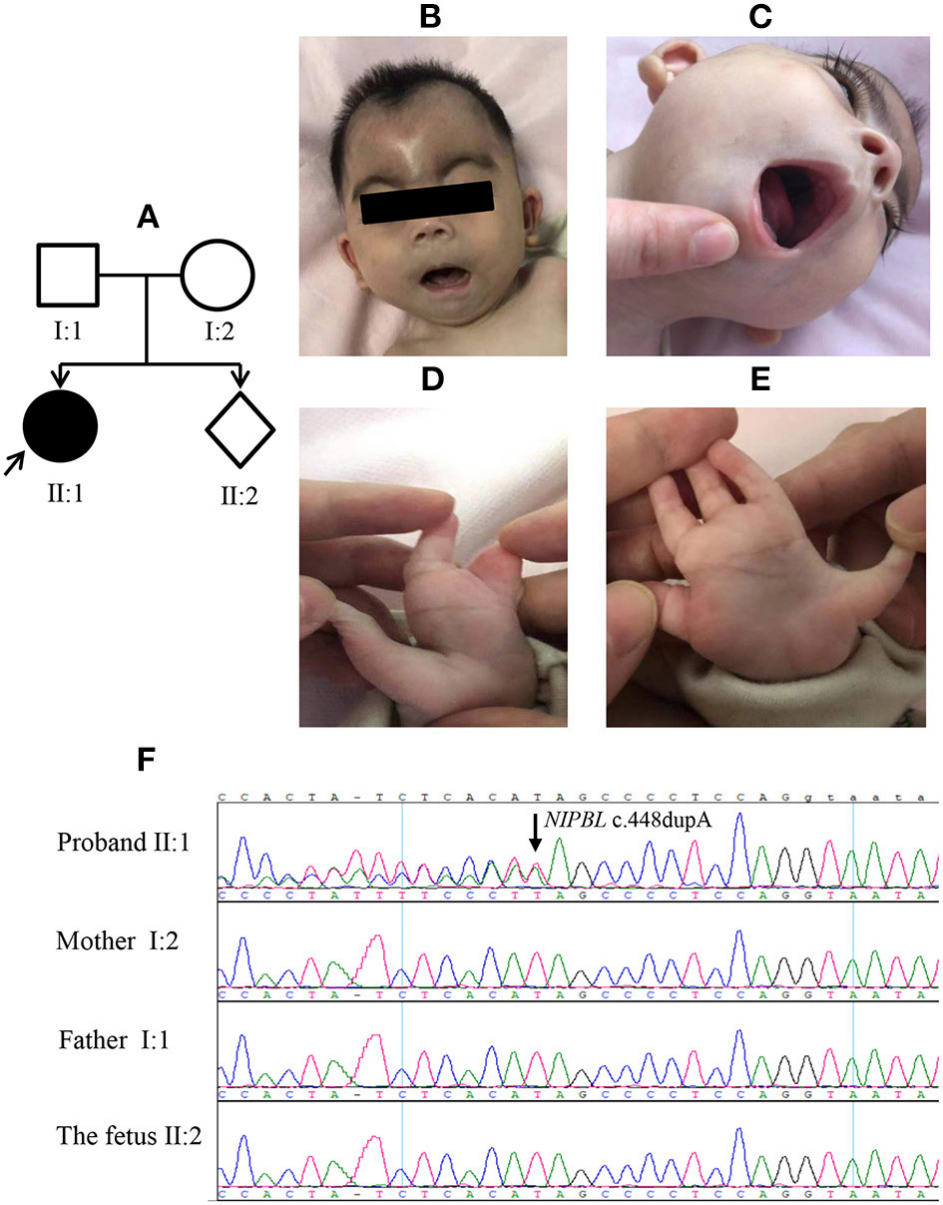
Clinical features of the proband and Sanger sequencing results in case 3. The proband **(A**, II: 1) presented with typical facial features of Cornelia de Lange syndrome (CdLS) and upper limber defects **(B–E)**. The **(F)** panel shows the Sanger sequencing results of *NIPBL* c.448dupA mutation in the family.

The index patient (an 8-month-old girl) was the first child of the first pregnancy of a healthy non-consanguineous Chinese couple with no remarkable history in genetic diseases (Figure 3A). The proband was born with a length of 45 cm, birth weight of 2,450 g and head circumference of 31 cm (<−2 SD). She was smaller than gestational age. The mother (aged 20) had been at 17 weeks of gestation in her second pregnancy by the time she presented seeking for prenatal diagnosis. The proband in case 3 had typical facial features of CdLS (Figures 3B,C; Table 1) with limb defects (Figures 3D,E), being classified as classic CdLS (Supplementary Table 1). WES in the family trios identified a *de novo* frameshift variant of *NIPBL* (NM_133433.4: exon 5: c.448dupA: p.S150Kfs^*^23) in the proband, which was validated by Sanger sequencing (Figure 3F). This mutation was predicted to have nonsense-mediated mRNA decay effect as the truncation occurred at the fifth exon of the 47 exons of *NIPBL*. By using reverse transcription quantitative PCR (details of the methods are provided in the Supplementary Materials and Methods), we observed a reduction around 40% of the *NIPBL* mRNA in the peripheral blood leukocytes of the proband compared with the healthy mother (Supplementary Figure 4). Loss-of-function mutations of *NIPBL* associated with CdLS were previously reported, and the c.448dupA variation was present on biologically relevant transcript. Thus, rule PVS1 was applied according to the ACMG guidelines. The *de novo* mutation met rule PS2, as well as rule PM2 since this mutation was not listed in public databases including dbSNP(v147), gnomAD, and the Human Gene Mutation Database (HGMD, http://www.hgmd.cf.ac.uk/ac/index.php). PolyPhen-2/Mutation testing and SIFT (http://sift.jcvi.org) predicted that this variant was probably damaging with a score of 1.0 (rule PP3). Overall, this variant was classified as pathogenic (rules PVS1, PS2, PM2, and PP3). Sanger sequencing with genomic DNA from amniocytes showed that the c.448dupA mutation was not present in the fetus of the second pregnancy of the couple (Figure 3F).

## Discussion

CdLS is a multisystemic malformation syndrome recognized primarily on the basis of characteristic facial dysmorphism including long and thick eyelashes (99%), synophrys and hypertrichosis of the brows (98%), thin lips with down-turned corners (94%), a depressed nasal bridge with anteverted nares (85%), widely spaced teeth and micrognathia (84%), hirsutism (78%), and cutis marmorata (60%) (Kline et al., 2018; Li et al., 2020). A review of 53 CdLS pregnancies revealed that FGR, limb abnormalities, and distinct facial features are the most remarkable sonographic findings for the syndrome (Clark et al., 2012). However, a previous study showed the unsatisfactory performance of routine prenatal ultrasonography on detection of CdLS cases with major malformations (Barisic et al., 2008). Although FGR was nearly always present in CdLS fetuses, it represented a non-specific condition (Clark et al., 2012). Increased NT has been observed in some CdLS pregnancies. However, 46% of CdLS pregnancies had a normal NT (Clark et al., 2012). This suggests that NT as an indicator alone is not sensitive enough for prenatal screening of CdLS, but fetuses with increased NT, especially in association with FGR, require vigilant assessment. In the present study, we reported two CdLS pregnancies (case 1 and case 2), with one having fetal NT thickening at the first trimester and the other showing FGR at the third trimester (Table 1). Notably, the major ultrasound findings of these two cases were not directly linked to the diagnosis of CdLS. Since NT is increasingly measured as a routine parameter for first-trimester risk screening, more data on the relationship of NT values and CdLS will be of clinic significance (Hulinsky et al., 2005; Clark et al., 2012).

Of CdLS patients, 30%−46% were reported to have upper limb deficiencies, which often show asymmetric involvement (Jackson et al., 1993; Mehta et al., 2016). In a cohort of 378 individuals with CdLS, Mehta et al. observed a consistent pattern of laterality and symmetry involvement regarding the limb defects, with increased severity of right-sided limb in individuals who manifested asymmetric limb defects. They proposed a correlation between more severe limb defects and an increased risk of other structural anomalies and more severe behavioral outcomes (Mehta et al., 2016). In the present study, two out of the three cases (case 1 and case 3) were seen with limb asymmetric involvement, having the right side more severely affected than the left side. Our observation is in line with the previous finding of right-sided predominance of limb anomalies in CdLS.

To date, according to the Human Gene Mutation Database (accessed on March 30, 2021), a total of 482 diverse mutations in the *NIPBL* gene have been identified to be associated with CdLS. In this study, missense mutation, gross deletion, and small insertion were observed in three patients with CdLS, respectively. Previous studies showed that reduced expression or activity of NIPBL protein could lead to CdLS (Krantz et al., 2004; Tonkin et al., 2004; Hulinsky et al., 2005). In case 1 of the present study, the microdeletion encompassed the first 43 exons of the 47 exons of *NIPBL*, almost resulting in a null allele. Although it is currently unknown whether the mutant is expressed or not, our case may provide additional evidence that haploinsufficiency of *NIPBL* is a pathogenic mechanism of CdLS. In case 3 of this study, the c.448dupA mutation theoretically produces a prematurely truncated protein. Gillis et al. (2004) found that individuals with truncating mutations presented a more severe phenotype compared with patients with missense mutations or non-identifiable mutations. In the present study, we did observe that the c.448dupA mutation manifested more severe upper limber defects compared with the c.5566G>A (p.R1856G) mutation in case 2.

Germline mosaicism can be the pathogenic mechanism in certain cases where the affected children in a family carried the same *NIPBL* mutation, but the mutation was not detected in the parents' blood samples. (Slavin et al., 2012). Supporting this hypothesis, the heterozygous pathogenic *NIPBL* missense mutation c.7298A>G was found in the sperm of a father with multiple affected offspring, but not in his peripheral blood (Niu et al., 2006). Additionally, 23% of CdLS probands without detectable mutation in lymphocytes were found to carry a *NIPBL* mutation in buccal cells (Huisman et al., 2013). Furthermore, mosaic variants were found in 4 out of 13 (30.8%) patients negative for germline alterations (Krawczynska et al., 2019). In our study, all the variants appeared to be *de novo* mutations in the patients because they were absent in the blood of both parents. However, the possibility of germline mosaicism cannot be completely excluded. With regard to the second pregnancy of the couple in case 3 that was diagnostically excluded from the *NIPBL* c.448dupA mutation, follow-up ultrasound examinations were provided to the fetus, and no major anomalies were found.

In conclusion, we reported three cases of CdLS, each having its own clinical indication for prenatal diagnosis (increased nuchal translucency, fetal growth restriction, or family history) and mutation type of *NIPBL* (gross deletion, missense, or frameshift). Our study demonstrates that the SNP array is able to identify the microdeletions that span the *NIPBL* gene, highlighting a diagnostic value of this tool in CdLS. To the best of our knowledge, the *NIPBL* gross deletion and c.448dupA variants identified in this study had not been documented previously. These cases may serve as references for increasing our understanding of the pathogenic mutation spectrum of *NIPBL*. Our study provides clinical cases for further investigation on the pathogenesis and diagnosis of CdLS.

## Data Availability Statement

The datasets for this article are not publicly available due to concerns regarding participant/patient anonymity. Requests to access the datasets should be directed to the corresponding author.

## Ethics Statement

The studies involving human participants were reviewed and approved by Ethics Committee of Hunan Provincial Maternal and Child Health Care Hospital. Written informed consent to participate in this study was provided by the participants' legal guardian/next of kin. Written informed consent was obtained from the minor(s)' legal guardian/next of kin for the publication of any potentially identifiable images or data included in this article.

## Author Contributions

YP, CL, and HW designed the study. YP, WX, and CT wrote the manuscript. JP and JL performed the SNP array analysis. YP and SY interpreted the data of SNP array and qPCR. HX and JH performed the karyotyping and WES. YL performed the prenatal ultrasound examination. All authors contributed to manuscript revision, read, and approved the submitted version.

## Conflict of Interest

The authors declare that the research was conducted in the absence of any commercial or financial relationships that could be construed as a potential conflict of interest. The reviewer L-LF declared a shared affiliation with one of the authors, CT, to the handling editor at time of review.

## Publisher's Note

All claims expressed in this article are solely those of the authors and do not necessarily represent those of their affiliated organizations, or those of the publisher, the editors and the reviewers. Any product that may be evaluated in this article, or claim that may be made by its manufacturer, is not guaranteed or endorsed by the publisher.

## Supplementary Material

Supplementary Figure 1 SNP array revealed a *de novo* heterozygous microdeletion of 0.2 Mb on chromosome 5p13.2 in the fetus in case 1.

Supplementary Figure 2 Real-time quantitative PCR confirmed the *de novo* microdeletion spanning the first 43 exons of *NIPBL* in the fetus in case 1. NIPBL-1,-2 and-3 primer pairs were used to target the exons 26, 29 and 30, respectively. In addition to the family trios, two healthy controls were employed for this validation assay.

Supplementary Figure 3 Intra-uterine growth curve of the fetus in case 2.

Supplementary Figure 4 Real time quantitative PCR detected a reduction of *NIPBL* mRNA in the periphery blood leukocytes of the proband compared with the healthy mother in case 3. The *NIPBL* mRNA levels were normalized to the *GAPDH* mRNA levels of the same sample to determine the ratios. The ratios of the mother were arbitrarily set as 100%. Data are expressed as mean ± SD (*n* = 3, two-tailed Student's *t* test), ^**^*p* < 0.01.

Supplementary Table 1 Clinical classification scoring of the three cases of CdLS.
