# Supplementary figures and images for: Case Report: Novel NIPBL Variants Cause Cornelia de Lange Syndrome in Chinese Patients
Source: Front Genet. 2021 Jul 30;12:699894. doi: 10.3389/fgene.2021.699894 (PMC8362598; doi:10.3389/fgene.2021.699894)

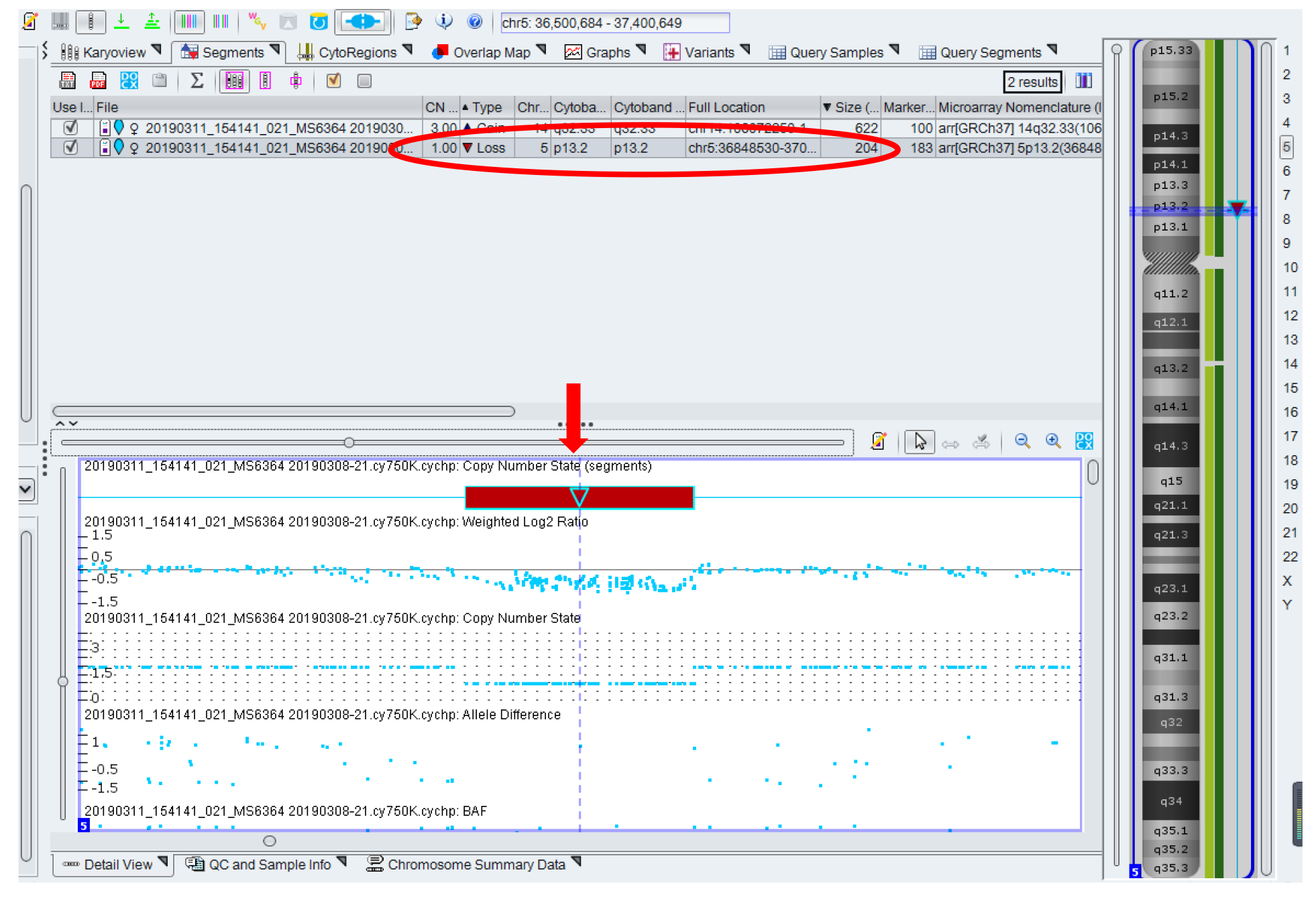

Supplement: Supplementary Figure 1 — SNP array revealed a de novo heterozygous microdeletion of 0.2 Mb on chromosome 5p13.2 in the fetus in case 1. [file Image_1.TIF]

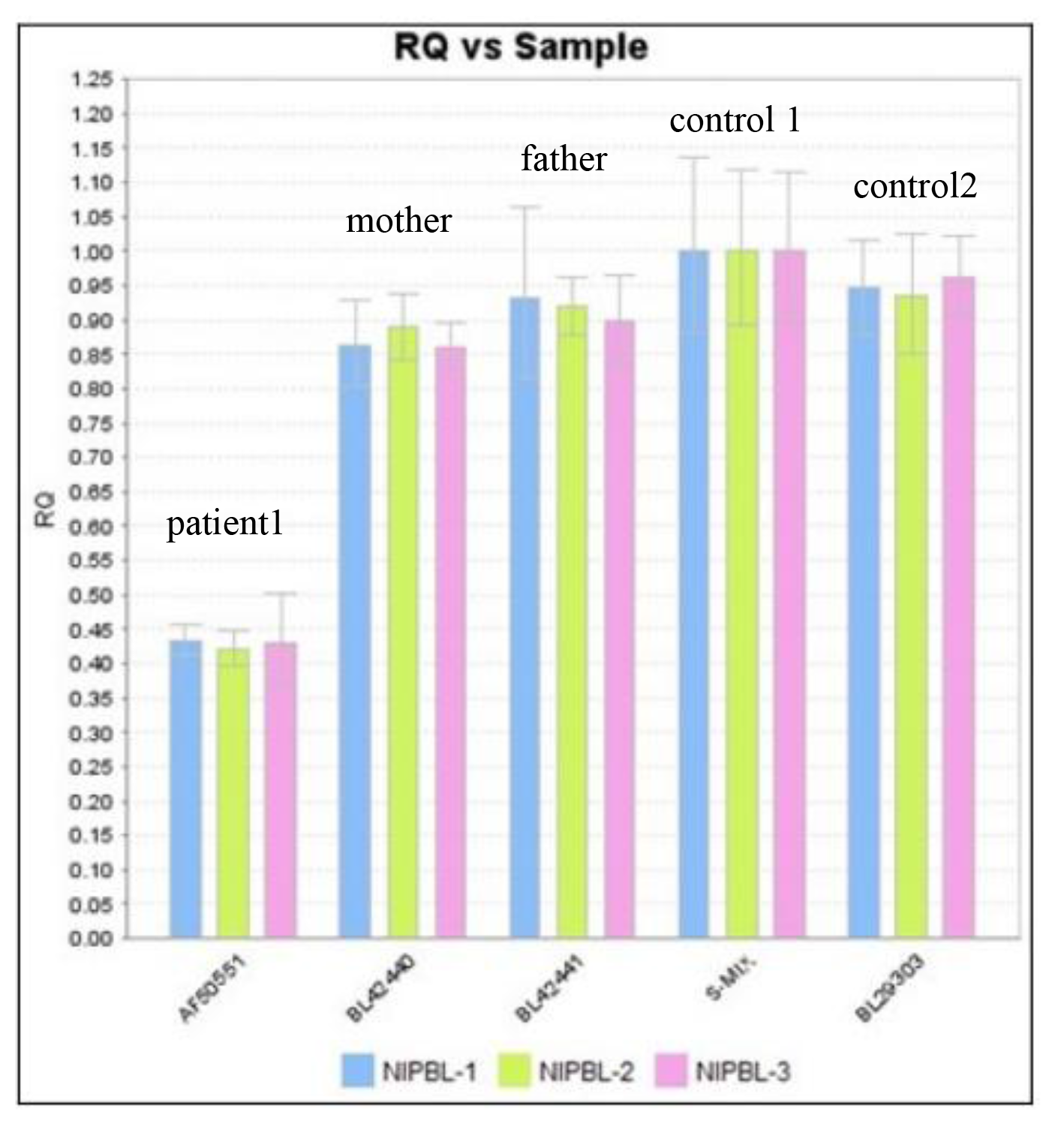

Supplement: Supplementary Figure 2 — Real-time quantitative PCR confirmed the de novo microdeletion spanning the first 43 exons of NIPBL in the fetus in case 1. NIPBL-1,-2 and-3 primer pairs were used to target the exons 26, 29 and 30, respectively. In addition to the family trios, two healthy controls were employed for this validation assay. [file Image_2.TIF]

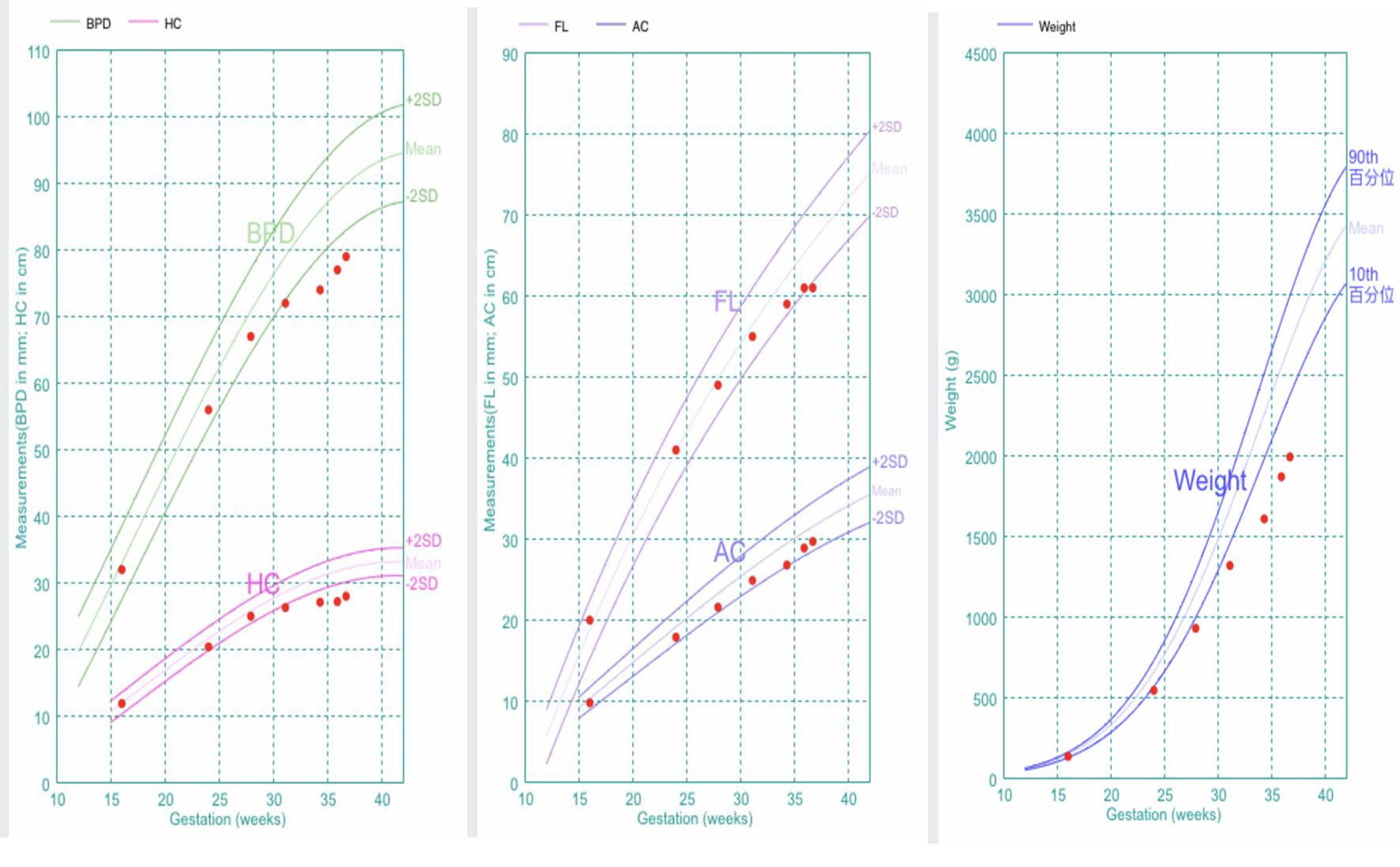

Supplement: Supplementary Figure 3 — Intra-uterine growth curve of the fetus in case 2. [file Image_3.TIF]

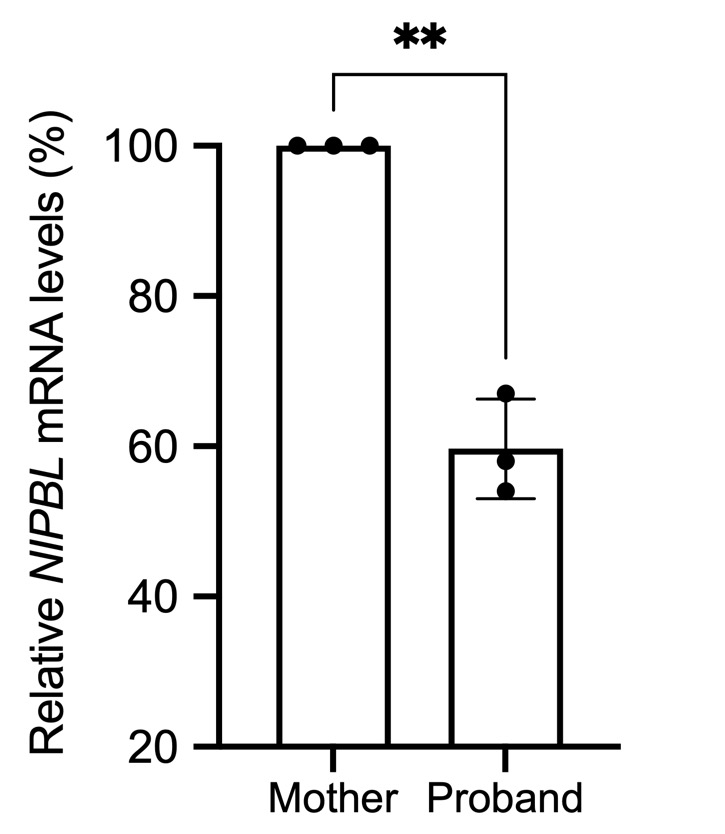

Supplement: Supplementary Figure 4 — Real time quantitative PCR detected a reduction of NIPBL mRNA in the periphery blood leukocytes of the proband compared with the healthy mother in case 3. The NIPBL mRNA levels were normalized to the GAPDH mRNA levels of the same sample to determine the ratios. The ratios of the mother were arbitrarily set as 100%. Data are expressed as mean ± SD (n = 3, two-tailed Student's t test), **p < 0.01. [file Image_4.TIF]
