# Supplementary material for: Case Report: Novel NIPBL Variants Cause Cornelia de Lange Syndrome in Chinese Patients
Source: Front Genet. 2021 Jul 30;12:699894. doi: 10.3389/fgene.2021.699894 (PMC8362598; doi:10.3389/fgene.2021.699894)
Supplement: Supplementary Table 1 — Clinical classification scoring of the three cases of CdLS. [file Table_1.docx]

Supplementary Table 1. Clinical classification scoring of the three cases of CdLS

| Clinical features | Case 1 | Case 2 | Case 3 |
| --- | --- | --- | --- |
| *Cardinal features (2 points each if present)* | | | |
| Synophrys and/or thick eyebrows | 2 | 2 | 2 |
| Short nose, concave nasal ridge and/or upturned nasal tip | 2 | 2 | 2 |
| Long and/or smooth philtrum | 2 | 2 | 2 |
| Thin upper lip vermilion and/or downturned corners of mouth | 2 | 2 | 2 |
| Hand oligodactyly and/or adactyly | 2 | / | 2 |
| Congenital diaphragmatic hernia | / | / | / |
| *Suggestive features (1 point each if present)* | | | |
| Global developmental delay and/or intellectual disability | / | 1 | 1 |
| Prenatal growth retardation | 1 | 1 | / |
| Postnatal growth retardation | / | 1 | 1 |
| Microcephaly (prenatally and/or postnatally) | 1 | 1 | 1 |
| Small hands and/or feet | / | / | 1 |
| Short fifth finger | / | / | 1 |
| Hirsutism | / | 1 | 1 |
| *Clinical score** |  |  |  |
| Total points | 12 | 13 | 16 |
| Classification | Classic | Classic | Classic |

*According to the first international consensus statement (Kline et al., 2018), a score of ≥11 points with at least 3 cardinal features indicates classic CdLS. The slash stands for absence of the symptom or not applicable for scoring.
