# Supplementary material for: Case Report: Novel NIPBL Variants Cause Cornelia de Lange Syndrome in Chinese Patients
Source: Front Genet. 2021 Jul 30;12:699894. doi: 10.3389/fgene.2021.699894 (PMC8362598; doi:10.3389/fgene.2021.699894)
Supplement: Supplementary file 6 [file Data_Sheet_1.docx]

Supplementary Materials and Methods

Total RNA from peripheral blood leukocytes was extracted with TRIzol reagent (Cat#15596026, Thermo Fisher Scientific) according to the manufacturer’s instructions. Complementary DNA was synthesized using reverse transcription reagents (Cat# N8080234, Thermo Fisher Scientific). Quantitative real-time PCR was performed with the TB Green Premix Ex Taq II reagent (Cat#RR820B, TaKaRa) on LightCycler96 Real-Time PCR System (Roche Life Science). For quantification, the *NIPBL* mRNA levels were normalized to the mRNA levels of *GAPDH*. Primers used were as follows: human *NIPBL*-forward 5′-GGCATGACTGTAGTGCAAC-3′, reverse 5′-ATTGAAACAAGCCCACACAA-3′; human *GAPDH*-forward 5′- CCAGAACATCATCCCTGCCT -3′，reverse: 5′-CCTGCTTCACCACCTTCTTG -3′.
